# Stability of Rowing Technique and Specificity of Training Load: A Pilot Longitudinal Study in Young Athletes

**DOI:** 10.3390/sports14050214

**Published:** 2026-05-21

**Authors:** Igor E. Anpilogov, Nicolas H. Kruchynsky, Eugene B. Postnikov

**Affiliations:** 1 Department of Biomedical Studies, Recreational and Adaptive Physical Education, Kursk State University, Radishcheva St., 33, 305000 Kursk, Russia; anpilogov.sport@yandex.ru; 2 Department of Physical Rehabilitation and Sport Medicine, Polessky State University, Dneprovskoj Flotilii St., 23, 225710 Pinsk, Belarus; nickolasha57@gmail.com; 3 Department of Theoretical Physics, Kursk State University, Radishcheva St., 33, 305000 Kursk, Russia

**Keywords:** training process, biomechanics, cyclic motions, sensors, rowing, inertial measurement unit, stroke stability, longitudinal study

## Abstract

Tracking biomechanical changes associated with different training modalities remains a methodological challenge in applied sports science. This pilot longitudinal study examined stroke technique stability in seven junior rowers (aged 16.6 ± 0.5 years) across three measurement sessions (March, April, June), separated by two training mesocycles emphasising strength training and intensive rowing, respectively. Upper body angular velocity was recorded using a smartphone-based MEMS sensor fixed to the upper back during incremental ergometer exercise. Overall stroke duration and its standard deviation remained stable throughout the study period, whereas the durations of the two stroke phases corresponding to forward (drive) and backward (recovery) body motion changed systematically across mesocycles. Phase-specific changes were statistically significant in 10 of 12 paired comparisons (rank-sum test) and 7 of 12 within-subject comparisons (Wilcoxon signed-rank test) for phase durations, and in 9 and 5 of 12 comparisons for their standard deviations, respectively. These findings suggest that the internal structure of the rowing stroke is sensitive to training load specificity, even when overall stroke timing remains unchanged, and that smartphone-based angular velocity analysis provides a feasible tool for individualized biomechanical monitoring in young athletes.

## 1. Introduction

Successful rowing performance crucially depends on an interconnection between physical, technical, tactical, and psychological preparation. Analysis of research results shows that rowers to achieve high competitive performance, along with high levels of strength, endurance, and coordination, must also be able to effectively transform their physical readiness into technical efficiency [1,2,3,4].

At the same time, the majority of studies in the field of rowing are focused on evaluating either sportsmen’s physiological indicators or biomechanics of rowing considered from the perspective of boat motion (see e.g., [5,6]). But research aimed at assessing the dynamics of an athlete’s own movement technique under varying power loads, increasing fatigue, and the effects of different types of training loads are more scarce [4,7]. Among the works related to the topic of the present study, one can mention exploring changes in the joint motion angles at the initial and final stages of a trial [8], coordination of motions of different extremities and its variability [9].

Following the growing use of inertial measurement units (IMUs) for quantifying the spatiotemporal characteristics of athletes’ movements [10,11,12], these sensors have been increasingly applied in rowing biomechanics research. Such studies have examined angle–time dependencies during rowing [13], joint coordination [8], and torso and pelvic inclinations [14], among other aspects [11,15,16].

But the majority of works [7,8,14,17] is primarily focused on assessing body segment angles to improve technique and enhance rowing performance, as well as on validating the reliability of IMU sensors and their ability to detect changes associated with variations in stroke specificity due to fatigue. However, the range of body angular inclinations is only indirectly related to the rowing phases corresponding to forward and backward body motions, as they are indicated by the derivative of the angle, i.e., the angular velocity. This limits the quantitative characterization of the process when only angles are addressed. At the same time, it is important to note that the physical principles underlying IMU operation [18] are based on measuring acceleration and angular velocity, while the angles themselves are obtained as integrated quantities. This suggests that direct analysis of the raw recorded signals may provide a more detailed characterization of movement variability. Such an opportunity has been explored in our previous works [19,20], which demonstrated a clear distinction between levels of rowing proficiency.

This is associated with the need for a more detailed exploration of the variability of sports motions during their execution as a part of the general problem in cyclic sports or sport branches [21,22,23,24] determined by various factors: the structure and complexity of the training process, fatigue during the execution of sport-specific periodic movements, the overall technical preparedness of the athlete, etc. These features of technique dynamics encompass a wide range of factors: variability of the stroke length and the ratio of their phases exhibited by differently skilled rowers [9,25], statistical comparison of different cervical spine angular biomechanical parameters [26], relations between movement speed and power output during rowing at different stroke rates [27], and, in general, variability detection and assessment by different measuring tools [28]. Also, it is worth noting that the majority of the studies cited above involve adult rowers, whereas young athletes may be more responsive to the specificity of training load, especially under conditions where its type is altered.

In this work, we focus on the concept of stability, defined in terms of changes in several indicators considered in complex. These include: (i) the average stroke periods within a series of successive trials with increasing loads, as well as within each individual trial grade considered for the full stroke’s period and its phases of forward and backward motions; (ii) variability in the standard deviation from the mean for these three durations; (iii) variability in the average standard deviations from the mean instant profile of the upper body’s angular velocity visualized as scattering of time-dependent curves and quantified numerically by the trial-averaged point-wise ratios of the instant standard deviation from the mean trajectory to the instant absolute values of the latter. The third approach requires high-resolution records of the instantaneous time evolution of mechanical variables associated with the whole periodic motion of an athlete’s body during rowing. In contrast to two-point parameters like periods of a stroke and its phases, these whole curve-based characteristics remain insufficiently explored in the literature.

Additionally, the training of skilled rowers is of interest not only for assessing the instantaneous level of technical proficiency but also for tracking its development over the course of longitudinal training and for establishing technical tools for its monitoring [29,30,31]. Therefore, in this work, we aimed to use the technical approach developed previously [19] to investigate the dynamics of body motion during rowing across three measurement stages separated by three months with a special focus on data features indicating stability of cyclic motions. More specifically, we aimed to explore whether changes in the temporal parameters of stroke technique could be identified simultaneously with the recordings of different training modalities under conditions of increasing sequential power load for several test sessions separated by the training mesocycles with different content of the training load.

## 2. Materials and Methods

### 2.1. Research Design

The study is designed as a longitudinal investigation involving three sequential stages of kinematic measurements, separated by month-long training mesocycles, of dynamic features exhibited by the same group of participants. These IMU-based assessments are considered in conjunction with information on the structure of training loads, which were identical for the whole group between measurement stages. Thus, given the relatively small number of participants and the uniformity of the group (young athletes), this study should be regarded as a pilot investigation of whether changes in factors characterizing rowing stability can be detected following modifications in the content of the training load.

### 2.2. Participants

The group under study included seven young sportsmen (aged 16.6 ± 0.5 years) specializing in rowing at Sport School of Polessky State University. Their physical and performance characteristics are provided in Table 1; all participants belong to Tier 3 (Highly Trained/National Level) according to the Training and Performance Classification [32]. Criteria of their inclusion into the study: (a) active participation in regional-level competitions, (b) no musculoskeletal injuries in the last 6 months, (c) regular use of a Concept2 ergometer. The anthropometric profile of the group corresponds to established predictors of performance in junior rowing [33]. At the time of the study, the athletes were in the preparatory phase of the macrocycle, performing 5–6 training sessions per week. To operate with anonymized data, they are numbered by separate Roman numerals in what follows. Before the study, all subjects provided written informed consent with approval from their legal guardians. This study was conducted in accordance with the guidelines of the Declaration of Helsinki and was approved by the Institutional Ethics Committee of Polessky State University (protocol No. 1 dated on 27 February 2025).

### 2.3. Experimental Protocol

The study protocol included graded exercise performed on an indoor rower (ergometer) Concept2 Model E (Morrisville, VT, USA) until a participant reached a blood lactate concentration of 4 mmol/L, following an incremental exercise test approach focused on the gradual increase of the lactate endurance marker [34,35]. Each graded exercise bout lasted 4 min, with 1 min rest intervals between them. The power load on the indoor rower started at 110 W and was sequentially increased by 40 W per stage. The initial power load was set to ensure a stable aerobic effort below the first lactate threshold and to clearly determine the onset of blood lactate accumulation in the young athletes, while the stepwise increase made it possible to obtain several data points to trace lactate accumulation over successive stages of the test, in accordance with the protocols mentioned above. The lactate concentration was determined using the portable analyzer Lactate Pro 2 (Arkray Global Business, Inc., Kyoto, Japan). As a result, three grades constituted the minimal number completed by all participants before reaching the threshold lactate level. This made it possible to obtain a homogeneous, balanced dataset in which all participants reached the same maximal lactate level and to avoid bias associated with differing degrees of fatigue among them. These three successive grades were selected from the stages of the study carried out in March, April, and June. Accordingly, the training process during the study period was divided into two mesocycles between the testing stages. The first mesocycle lasted six weeks and corresponded to the training period between the first and second testing stages. The second mesocycle comprised nine weeks of training between the second and third stages of the study.

The overall training load was assessed based on the analysis of athletes’ individual training diaries. Records describing the quantitative content of each training session were entered into the diary immediately after its completion. They included the following: strength exercises counted by the total mass of an external weight lifted; continual rowing using an indoor rower counted by the distance covered; interval rowing using an indoor rower counted by the sum of the distances covered by an athlete rowing at high intensity; the same, principally, quantities for on-water rowing with the difference that since on-water rowing was performed along an unmarked river course, the on-water interval rowing was counted as the sum of the time intervals (in minutes) spent by an athlete carrying out rowing at high intensity. To ensure data reliability, all records were cross-checked weekly by the coach and the lead researcher. Any discrepancies were resolved through immediate follow-up interviews with the athletes.

### 2.4. IMU Measurements and Data Processing

Following the approach developed and described in the works [19,20], measurements of time series representing the angular velocity of a participant’s upper back were carried out using the MEMS (micro-electromechanical system) sensor of the smartphone Honor FNE-NX9, fixed with a special chest-mount harness, and the ‘phyphox’ software [36], wirelessly paired with a PC. The device-conditioned time sampling rate is 0.001 s. The ability of the angular velocity recorded under such experimental conditions to characterise athletes’ rowing performance has been analysed in detail and confirmed in the work [19].

The angular velocity data, saved in text format, were processed with a home-written MATLAB R2014b code as follows. At the first step, the time series with the discrete time step 0.001 s were smoothed by cutting off the high-frequency part of their Fourier spectra exceeding the frequency *f*_cut-off_ = 2.23 Hz. This cut-off frequency is determined by the following considerations: this value corresponds to a period equal to 0.45 s, while the typical period of a full stroke is *T* = 2–4 s. Hence, this cut-off preserves not less than four higher harmonics of the periodic signal under investigation. As analyzed in detail in [19], the principal features of the angular velocity curve shape during rowing are mainly determined by the fundamental and two higher harmonics (the second and the third). Therefore, the applied cut-off eliminates noisy random fluctuations while preserving the variability of individual strokes.

The obtained curves, reconstructed via the inverse Fourier transform, were segmented into datasets corresponding to individual strokes using a zero-crossing criterion, i.e., transitions of the recorded angular velocity from negative to positive values between successive data points. These sign changes reflect the alternation between moving forward and backward body tilts, corresponding to the catch–drive phase and the recovery phase, respectively [37]; see also the comparison of sequential photos and recorded curves in [20].

For the aligned subsets of individual stroke data, mean values and standard deviations of the time series were computed, including total stroke durations (i.e., stroke periods) and the durations of their phases, using standard MATLAB statistical functions. Additionally, given the small sample size and the absence of an a priori assumption of normality, median values were compared as more robust estimates of central tendency using a rank-sum test. It should be noted that this test was employed as a nonparametric procedure for median comparison, rather than as a test of distributional equivalence (as in the Mann–Whitney test, which formally relies on the same mathematical framework but assumes independent samples). To statistically assess the effect of mesocycle training on stroke duration-related indicators, the Wilcoxon signed-rank test was applied, as it is appropriate for comparing two dependent measurements obtained from the same subjects (i.e., pre- vs. post-intervention within the group). Both the rank-sum and signed-rank tests were implemented using standard MATLAB functions.

As an additional test operating with time series representing the full course of a stroke, we also applied a new criterion of stability defined as follows:

(1) $$\langle \frac{\mathrm{STD}(\omega_{x})}{|\omega_{x}|}\rangle =\frac{1}{\langle T\rangle }\int_{0}^{\langle T\rangle }\frac{\mathrm{STD}\left(\omega_{x}\right)\left(t\right)}{\left|\langle \omega_{x}\rangle \right|\left(t\right)}dt.$$

Here, $\left|\langle \omega_{x}\rangle \right|\left(t\right)$ is the function representing the instant absolute value of the mean angular velocity, and $\mathrm{STD}\left(\omega_{x}\right)\left(t\right)$ is the function giving the point-wise standard deviations of the velocity time series for individual strokes from the mean curve. The integral is taken over the average period of strokes with three excluded points corresponding to $\langle \omega_{x}\rangle =0$ (two boundary points and one point inside of the integration interval). Numerical calculations, including the trapezoidal rule of integration, were implemented using standard MATLAB functions.

## 3. Results

### 3.1. Changes in Rowing Technique Time Parameters

Since the proposed instrumental method allows for producing visualized tracks of angular velocity that illustrate continuous variations in the time series of rowing dynamics, the presentation of the obtained results is organized as follows. First, we present figures containing examples of the recorded data and their typical features. After that, the numerical data, based on these time series and organized for the entire group of participants, will be reported.

**Figure 1:**
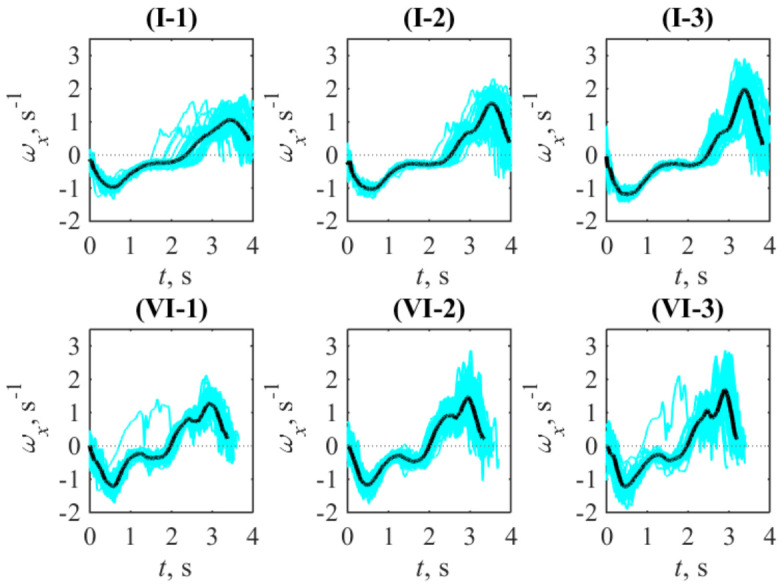
Examples representing superposition of the angular velocity time series for individual strokes (cyan curves) and their average value (black curve) for three graded exercise (numbered by Arabic numerals) in the case of two participants (numbered by Roman numerals). The data were recorded during the second stage of exercises (in April) and illustrate how the upper body’s angular velocity varies from stroke to stroke in comparison with the average time course for different grades of power load.

Figure 1 shows representative angular velocity time series recorded during graded ergometer exercise for two participants across three power load grades. These examples illustrate the typical features of data visualized as curves. First of all, this cyclic motion is asymmetric and can be conventionally subdivided into two phases, coordinated with the specificity of rowing technique: the drive (phase 1) and the recovery (phase 2). Phase 1 has negative angular velocity and corresponds to the case when the athlete overcomes the load created by the ergometer. Phase 2 is characterized by positive angular velocity and corresponds to the situation when the athlete’s body returns to the initial position without external force loading.

Qualitatively, the scattering of angular velocity is seen in the variety of cyan curves in Figure 1, which are dispersed around their instantaneous mean values (the black curve) to different degrees. One of the visible patterns corresponds to the case in which the increase in power load (indicated by the increasing grade number) is associated with improved stability, as illustrated by participant I: at grade 1, large deviations of individual stroke trajectories from the mean stroke are observed in phase 2; at grade 2, the trajectories of individual strokes become smoother; and at grade 3, the smallest deviations of individual strokes from the mean stroke profile are recorded. Another pattern has also been identified: in the case of participant VI, at grade 1, there are substantial deviations of individual strokes in phase 1; as power increases, these deviations are reduced at grade 2, but at grade 3 of the exercise, the magnitude of deviations of individual strokes increases again.

**Figure 2:**
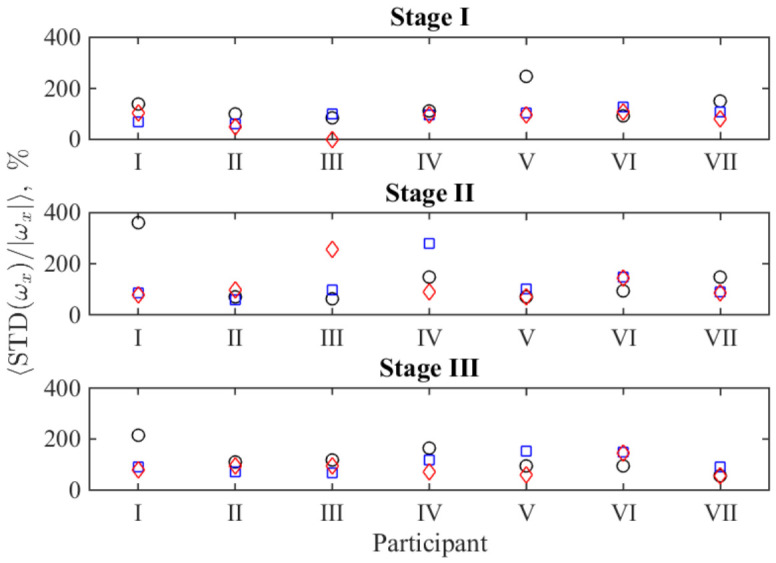
Overview of the average instantaneous relative scattering of the time course of the upper body’s angular velocity during the rowing process for all participants included in the study. Black circles, blue squares, and red diamonds denote the first, second, and third grades of sequential power load, respectively.

Figure 2 demonstrates the results of the calculations characterizing the instant stability of motion in a quantitative way, according to the definition given by Equation (1). They are provided for all participants, grades and stages, and visualized by markers; these relative quantities are expressed as percentages. With respect to the graphical representation in Figure 1, the functions $\left|\langle \omega_{x}\rangle \right|\left(t\right)$ and $\mathrm{STD}\left(\omega_{x}\right)\left(t\right)$ in Equation (1) correspond to the black and cyan curves in Figure 1.

**Figure 3:**
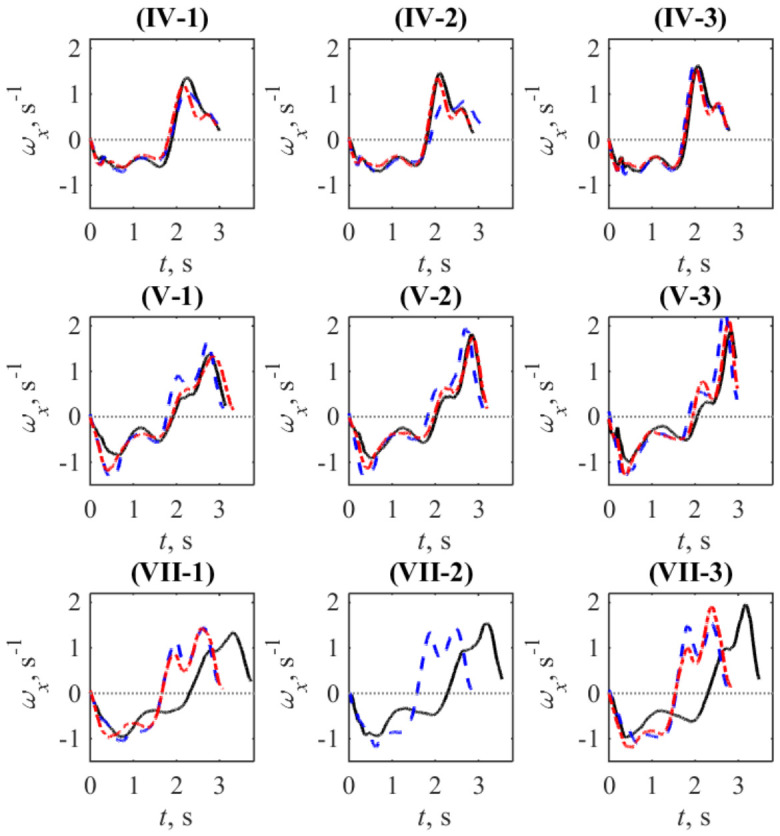
Examples of the average angular dynamics exhibited by three participants (numbered by Roman numerals) within three graded exercises (numbered by Arabic numerals) characterized by the sequential growth of the power load during three stages of study (March: black solid curves; April: blue dashed curves; June: red dash-dotted curves).

In addition to analyzing full curves representing strokes, we also examine variability in the average duration of a stroke and its phases. Examples of this phenomenon for individual participants are shown in Figure 3, where the curves of the average angular velocity for different stages are superposed.

In the case of participant IV, the stroke time changed visibly changed only at the second grade (see the shift of blue dashed curve) after the first mesocycle; there are no significant changes in stroke time between the first and third stages. In the case of participant V, visual changes in stroke time were observed at the second stage of the study (April) at all grades of the test exercise. A representative example of a drastic stroke-time change is shown in the subplots of Figure 3 related to participant VII.

To complete the qualitative examples with the quantitative values for the whole group of sportsmen under study, we provide a table with all individual numerical characteristics in Supplementary Materials.

It is worth noting that a change in stroke time by itself does not explain how an athlete manages to perform the stroke faster. For this reason, we analyzed the temporal parameters of the individual stroke phases too. Table 2 reports mean durations of all respective time intervals and their standard deviations as indicators of the technical stability, averaged over the whole group of participants.

To quantify the effects of training load on stroke parameters and their phases, complementing the curve-based discussion in the previous subsection, we applied two nonparametric rank tests to data computed for the participant group: the durations of rowing motions, which characterize temporal variability, and their standard deviations, which reflect stability in a stricter sense. The results are reported in Table 3. The rank-sum test was used for pairwise comparisons of mean durations and standard deviations between months separated by one or two training mesocycles, as a nonparametric method for comparing medians of two datasets. The Wilcoxon signed-rank test was used to assess the significance of changes within the same group by comparing ranked measurements obtained before and after the intervention.

Table 3 shows a large number of significant changes, indicated by the number 1 in the table’s cells, at the chosen significance level when comparing different stages of rowing sessions separated by mesocycles. In more detail: all data in the column entitled ’Full stroke’ have the format 0/·, where 0 denotes the absence of significant changes at the level p=0.05. On the contrary, the durations of individual phases demonstrate such changes: in the columns ‘Phase 1’ and ‘Phase 2’ of Table 3, the rank-sum test for group-based changes indicates 10 of 12 significant occurrences (denoted as 1/·) in paired comparisons of group-based medians and 7 of 12 occurrences for paired pre/post responses within the same participant sample, quantified using the Wilcoxon signed-rank test. They are detected when two sequential mesocycles (March→April and April→June) are compared.

For the average stroke periods, the respective comparison has the form ·/0, except for one case only (Grade 1, the comparison of the April and June stages). However, this difference should be interpreted with caution because of the relatively small sample size. For individual phases, 9 of 12 cells in Table 3 are formatted as ·/1 for the rank-sum test, and 9 of 12 cells show the same result for the Wilcoxon signed-rank test. Again, the difference between March and June is less pronounced.

### 3.2. Changes in Training Load During the Study Period

**Figure 4:**
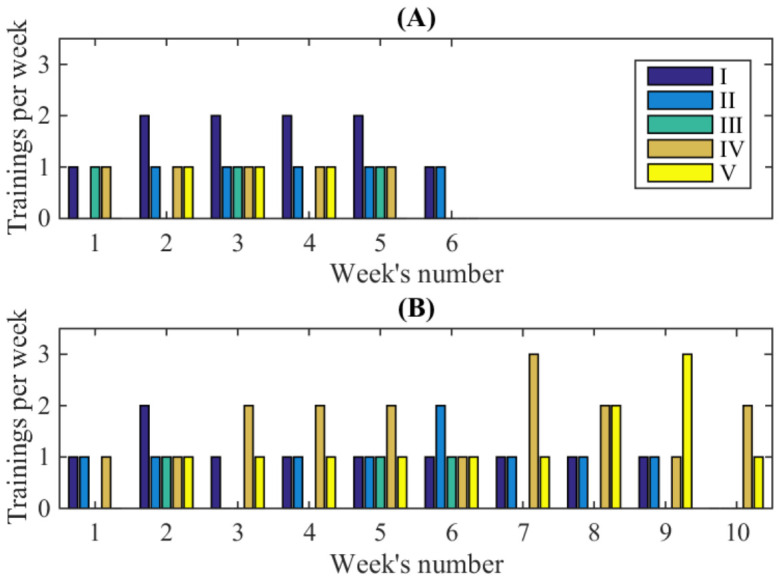
Training loads during the first (**A**) and the second (**B**) mesocycles. The structures of trainings indicated by the colour legend, the same for both mesocycles: I—Strength exercises; II—Continual rowing using an indoor rower; III—Interval rowing using an indoor rower; IV—Continual rowing on water; V—Interval rowing on water.

The dynamics of the temporal parameters of rowing technique over the entire study suggest that one factor influencing the time spent rowing is the training load performed between testing sessions. The analysis of the athletes’ training diaries made it possible to reveal the quantitative characteristics of the training loads summarized in Table 4, and the dynamical distribution of training during two mesocycles shown in Figure 4. Detailed information on the specific distribution of each type and volume of exercise across the days of each month in the training mesocycles is reported in Supplementary Materials.

## 4. Discussion

### 4.1. Training-Induced Changes in Dynamics of a Stroke and Its Phases

This study aimed to investigate whether changes in training modalities can be reflected in objectively registered changes in rowing technique, primarily in the stability of stroke repetitions during sequential rowing intervals performed on a rowing machine (ergometer) with increasing power load. The primary interest is the comparison between results obtained under different power loads on the ergometer during recording sessions separated by the training mesocycles.

The topic of stability involves consideration of three indicators.

**Figure 5:**
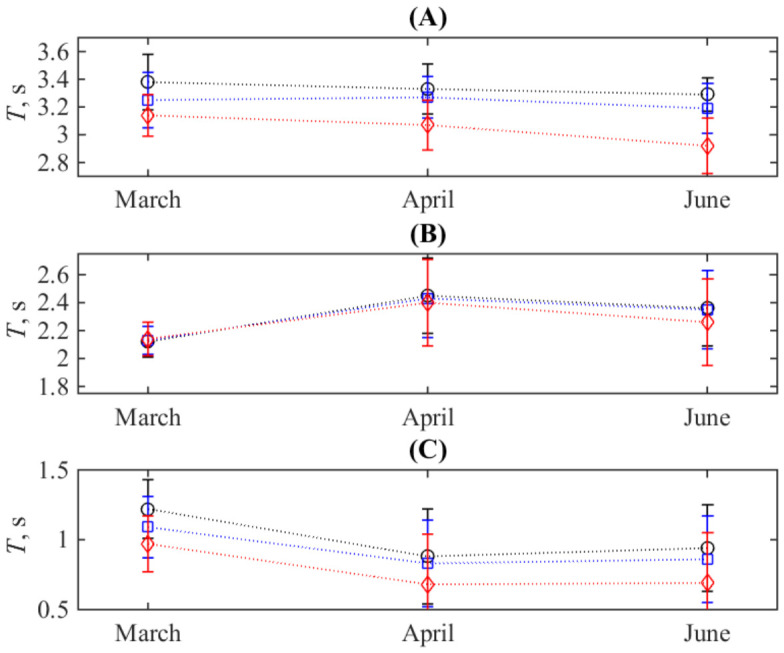
The stroke duration averaged over the whole group of participants for the whole stroke duration (**A**), its first (**B**), and the second (**C**) phases. Black circles, blue squares, and red diamonds denote the first, second and third sequential graded exercise. Whiskers are the standard deviations of these quantities. Dotted lines are added for visual guidance.

The first one is the average stroke duration and the durations of its two phases. On the group-averaged level, as illustrated in Figure 5A, the change of a full period has a weakly expressed decrease during the three recording sessions, which is comparable to the size of the whiskers denoting the standard deviations. On the contrary, visual deviations of the markers from a straight line can be traced for durations of a stroke’s phases, see Figure 5B,C for two sequential mesocycles (March→April and April→June). The long-term changes between the March and June data are less pronounced. This observation is supported by the results of the statistical tests presented in Table 3.

The second indicator, the standard deviation of the durations of a full stroke and its phases, which characterizes the stability of stroke performance at the group level, exhibits a similar pattern when comparing changes between mesocycle-separated records. Again, the difference between March and June is less pronounced.

The third indicator, $\langle \mathrm{STD}(\omega_{x})/|\omega_{x}|\rangle$, combines both factors for considering a stroke as a whole. Its variability across the group of participants (see Figure 2), allows additional arguments for discussing the features revealed at the group level. In particular, one can see that the circle markers corresponding to the lowest power load (grade 1) are most often positioned far from the markers corresponding to grades 2 and 3. As seen in Table 3, the significant changes in the factors listed there are also most pronounced for grade 1. The overall scattering of markers shown in the subpanel corresponding to stage 2 is visibly higher than that in the subpanels corresponding to stages 1 and 3. This is also consistent with the noted larger pairwise differences between two subsequent stages separated by one mesocycle than between the initial and final stages. At the level of individual participants, the numerical values represented by the markers in Figure 2 are consistent with the visual displacement and shape changes shown by the average curves in Figure 3. It is worth noting that the values depicted often exceed 100%, which follows from the fractional nature of the formula applied. Such unusually large values arise due to variability in the durations of the phases corresponding to the forward and backward body motions. Although the point $\langle \omega_{x}\rangle =0$, which separates these phases, is excluded from the calculations, the small average values $\left|\langle \omega_{x}\rangle \right|$ in its vicinity, together with the still large angular velocity in a particular individual stroke, result in a high value of the integrand and, subsequently, the integral as a whole. Thus, a large value of this indicator indicates high instability of the instantaneous rowing phases: their delay or advance relative to the average phase-transition time of the stroke.

The noted stage-paired changes in kinematic indicators can be discussed in comparison with changes in training content reflected in Table 4 and Figure 4. The dynamics of training load indicate an increase in the total volume of work from the first to the second mesocycle, accompanied by a slight decrease in the volume of strength training. Instead, different types of rowing are predominant in the second mesocycle.

Temporal parameters of the stroke make it possible to identify several trends extracted from Table 2. Phase 1 duration increased significantly in April across all load grades (12–14%), accompanied by a marked increase in standard deviation (from ±0.11–0.12 to ±0.27–0.30 s), indicating reduced timing stability. Conversely, Phase 2 execution time decreased substantially in April (24–30%) and only partially recovered in June, while its variability similarly increased (standard deviation up to ±0.31–0.36 s).

### 4.2. Plasticity of the Rowing Dynamics in Response to Training Load

The results of this study suggest that concentrating on specific training loads within defined time intervals may influence the specificity of rowing technique which can be regarded as a form of plasticity of oscillatory dynamics. Here, this term follows the terminology used in the mathematical theory of dynamical systems, where it denotes the adaptation of oscillatory (or rotational) phase responses to changes in the parameters of a system of coupled nonlinear oscillators (rotators) (see, e.g., [38,39,40]). In our case, we are focused on the phases of cyclic angular motions distinguished by the sign of the angular velocity. At the same time, this ’oscillatory phases’ correspond to ’phases of a stroke’ and can be related to neuromuscular adaptability at the bionic level [41]; however, a hypothesized physiological interpretation of the dynamical phenomena observed will be discussed in the next subsection.

This apparent plastic response is most clearly expressed in the interrelation between two principal phases of a stroke, which differ by either presence or absence of the external force load (in our case, provided by the ergometer) during the motion. In this context, the use of data from an IMU device, which directly records the angular velocity of an upper body motion, is straightforwardly useful since these phases differ by the sign of the angular velocity. Thus, one can make segmentation of time series easily and investigate the phase durations, their stability in timing quantified by the standard deviations of time intervals (see Table 2 and Table 3) and the stability of the time-distributed individual strokes quantified by the averaged instant standard deviations from the mean stroke profile (see Figure 2).

The concentration of strength training in mesocycle 1 (weeks 2–5) led to a marked change in the internal structure of the stroke. The observed increase in Phase 1 duration, together with the decrease in Phase 2, suggests that this phenomenon may originate from a shift in neuromuscular control strategies following the concept summarized in the book [42].

From the perspective of control theory as applied to rowing biomechanics [43], a specific strength load may temporarily alter movement coordination. In particular, the need to overcome high power loads may force the motor system to prioritize the physiological recruitment of high-threshold motor units at the expense of the technical smoothness of motion, i.e., by lowering its stability [44]. In this case, such a slowing of the active phase, when the rower is resisting the load generated by the ergometer, which is consistent with the findings reported in [45], suggesting that heavy strength work may temporarily hinder the development of the explosive speed of competition-specific movements, should be considered not merely as a mechanical prolongation of time but as a redistribution of physiological degrees of freedom aimed at stabilizing the output force [46].

In addition, we observe that a change in the median duration of Phase 1 is accompanied by a change in the corresponding median of standard deviations, that is, in one of the factors quantifying the stability of the timing of this phase. This effect is significant at the group level in the pure median (rank-sum) comparison and is also significant at Grade 1, the lowest ergometer power load, when the paired pre/post response within the same sample of participants is analyzed statistically. This feature may be interpreted as a “technical interference effect”, meaning that concentrated strength loads induce residual neuromuscular fatigue that disrupts the athlete’s ability to maintain a stable kinematic pattern during the high-intensity part of the stroke. The reduction in total stroke time and in the duration of Phase 1 indicates that rowing-specific exercises promote a better integration of power and speed.

The dynamic nature of Phase 2 (recovery) is also noteworthy. As training shifted from a strength-oriented to a speed-oriented emphasis, the prolongation of Phase 2 may reflect improved technical economy. According to several studies [47,48], the ability to prolong or stabilize the recovery phase without losing boat speed is a hallmark of elite proficiency, allowing more effective physiological recovery in each cycle. Our results suggest that Phase 2 is particularly sensitive to the training stimulus: while strength training may “compress” this recovery window and reduce its stability, speed work may contribute to a more relaxed and coordinated return to the starting position.

However, the pronounced variability observed among athletes, for example the contrast between participants IV and VII, highlights that group-level averages may mask critically important individual adaptation patterns. In light of these results, we believe that technical “maturity” in young athletes is nonlinear: some maintain stability under load, whereas others experience technical breakdown. This underscores the need for individual biomechanical monitoring. Relying solely on group averages risks overlooking athletes who may overtrain or fail to assimilate the technical demands of high-intensity work.

### 4.3. Consistency with Prior Findings on Training-Induced Kinematic Changes

The results of this study, indicating a potential relationship between specific training methods and the biomechanical structure of the stroke in young athletes, look consistent with some findings reported using alternative approaches, although it is worth noting that the following comparisons remain rather qualitative due to the pilot character of our study.

In particular, the assumption that variability of kinematic characteristics, considered as a dynamic response to the physiological and neuromuscular demands imposed by different mesocycles, can be qualitatively aligned with results reported in the work [49], indicating that the metabolic cost of rowing reflects an interplay between the drag force and the rowing speed. At the same time, it is mentioned that although rowing ergometer performance correlates with the strength of various body parts, extensive strength training does not exhibit a straightforward positive effect on the rowing technique and should be applied with caution. To some extent, it is aligned with our observations of the worsening of the stability indicators detected after the first (force-oriented) mesocycle of training.

The authors of the work [50], who also used a rowing simulator, reported evidence of non-uniform changes in the durations of stroke phases relative to the full stroke period in response to stroke effort. The physiological and biomechanical origins and details of this effect still need to be explored further; our results, illustrated, for example, in Figure 5, also support its existence. Although they are limited, we hope that the proposed use of angular velocity, which is a sensitive indicator for determining phase boundaries via the zero-crossing criterion, will shed additional light on this issue. Note that the importance of tracking the body segment velocities is also highlighted in the comprehensive book [43].

The redistribution of phase duration during periods of high-intensity strength work mentioned above suggests a possible change in neuromuscular strategy. The recent study [51], based on the machine-learning data processing for a complex of physiological and training intensity parameters, attracts attention to their entanglement affecting the rowing performance. It was noted that not only mean values of quantitative factors but also their fluctuations should be taken into account for improving the predictive capacity of such a class of models.

These observations pointed out the importance of a periodized approach to technical training, in which coaches should consider the balance between explosive strength development and the maintenance of kinematic stability. In particular, the analysis presented in [52] emphasized the importance of stable periodic stroke-phase sequencing in accordance with a chosen rowing technique model, alongside perceived force application. The cited study was based on visual evaluation by several experienced coaches. Thus, we can only note a certain qualitative similarity between patterns discussed in [52] and in our work.

### 4.4. Practical Coaching Implications

The proposed method for tracking changes in rowing technique requires only readily available tools: a smartphone running the free application ’phyphox’, which can be paired with a personal computer for real-time visualization and data storage. This makes it suitable for routine use in training and particularly advantageous for individualized monitoring. Within this context, it is worth paying attention to stroke-to-stroke shape variability, which is too rapid to be noticed by the naked eye but is easily detectable in the displayed instrumental records. Despite the limited amount of data processed in this work, we can hypothesize that a coach should give priority to monitoring the dynamics of both the overall stroke time and the durations of its individual phases when designing training blocks.

Thus, performing strokes at maximal loads is likely to be characterized by a decrease in stroke stability and an increase in the duration of phase 1 of the stroke, probably due to the accumulation of muscular fatigue. Conversely, an improvement in the coordination of the athlete’s actions during the stroke is likely to be accompanied by a lengthening of phase 2 against a background of stable overall stroke time. Therefore, maintaining a balance between explosive strength development and the preservation of kinematic precision of movement when planning training loads can be expected not only to ensure a nonlinear progression of young athletes’ technical mastery but also to minimize the risk of cumulative fatigue.

### 4.5. Limitations

Finally, we note the principal limitations of this study.

Although it has been tested earlier [19] that smartphone-based MEMS devices used as IMU devices provide sufficient accuracy to characterize rowing proficiency, it should be taken in mind that the reported numerical data have some uncertainty affecting quantitative measurements of the quantities discussed. Additional errors can be introduced by the positioning of a single sensor and the quality of its fixation on an athlete’s body [53].

The relatively small sample size implies that the observed patterns should be interpreted as indicative rather than strictly statistically conclusive in terms of their applicability to broader groups of athletes. It should also be noted that substantial effect sizes were observed only for several temporal parameters in individual athletes, which influences the statistics of such a small group. At the same time, the presence of these effects may still be useful for monitoring the skills of individual athletes, particularly since rowing is not a team game. In this context, we also acknowledge that other factors, such as individual maturation rates and cumulative fatigue, may contribute to the observed changes. This limits the strength of the interpretative conclusions that can be drawn from the detected variations of the rowing stability.

A certain limitation is also the homogeneity of the sample studied, which limits the generalizability of the present conclusions to athletes across broader age ranges and training levels without further investigation.

Another significant limitation of this study is its reliance on an indoor rowing machine and the lack of on-water validation against ergometer data. Although ergometers are indispensable for standardizing training loads, especially in regions where climatic conditions preclude on-water training from October to April, they do not fully reproduce the complex balance requirements, which strongly depend on the hydrodynamic effects of real boat rowing. As has been noted in a number of studies [54,55,56], the absence of a moving water surface and water resistance alters the acceleration–deceleration patterns of the kinematic chain [55]. However, considering that young athletes typically acquire their basic technical skills on rowing ergometers, and that the use of wearable sensors is well suited for monitoring rowing proficiency under these training conditions [17], we believe that our results provide valuable insights for this specific stage of skill development.

### 4.6. Future Research Directions

The phenomena observed in this work, as well as its limitations mentioned above, suggest several directions for future research: (i) to increase the sample volume by enriching the representation of athletes across different ages and levels of proficiency; (ii) to conduct investigations over longer time periods to accumulate more simultaneous data on changes in training load (supplemented with more detailed records of physiological parameters) and features of an athlete’s body mechanical dynamics, thereby enabling quantitative exploration of possible correlations based on time series using well-substantiated numerical data processing methods; (iii) to systematically investigate the effects of sensor placement on an athlete’s body in order to improve the sensitivity of recordings to typical rowing motions, as well as to explore the use of multiple sensors to develop a more comprehensive scheme for objective monitoring; (iv) to explore more sophisticated indicators of data variability, such as the dynamics of the instantaneous spectral content of angular velocity curves [19]; (v) to carry out the same kind of investigations during on-water rowing and evaluate the comparative analysis with the ergometer-based results.

## 5. Conclusions

In this work, we proposed the use of an IMU device based on a smartphone MEMS sensor, fixed on an athlete’s upper back, for the quantitative detection of changes in the time course of the body’s angular velocity during rowing.

Analysis of strokes across three measurement sessions, separated by two training mesocycles (the study spanned from March to June), revealed statistically significant differences in the ratio of average stroke phase durations and their stability. Simultaneous qualitative comparison with the structure of training loads during the mesocycles made it possible to hypothesize that the observed kinematic changes may be associated with the predominance of different types of training loads (strength training vs. intensive rowing) during the mesocycles preceding the recording sessions.

We believe that these phenomena, currently considered at the level of a pilot study, warrant further investigation involving a larger and more diverse group of participants, as well as a wider range of rowing and training conditions, as they may contribute to the development of a more balanced training process.

## Figures and Tables

**Table 1:** Physical and performance characteristics of the participants.

Variable	Mean ± SD	Range (Min–Max)
Age (years)	16.6 ± 0.5	16.0–17.0
Body mass (kg)	67.8 ± 4.2	62.0–74.0
Stature (cm)	174.4 ± 3.5	164.0–181.0
Rowing experience (years)	4.7 ± 0.8	3.0–5.0
2000 m ergometer time (min: s)	06:50 ± 0:08	06:40–07:05
Training volume (hours/week)	12.5 ± 1.5	10.0–15.0

The data in the second column are presented as (mean ± standard deviation (SD)).

**Table 2:** Time characteristics of a stroke over the group of athletes studied: mean durations of a full stroke and its phases supplied with standard deviations.

Stage	Grade	Full Stroke, s	Phase 1, s	Phase 2, s
March	1	3.38 ± 0.20	2.12 ± 0.11	1.22 ± 0.21
	2	3.25 ± 0.20	2.13 ± 0.10	1.09 ± 0.22
	3	3.14 ± 0.15	2.14 ± 0.12	0.97 ± 0.20
April	1	3.33 ± 0.18	2.45 ± 0.27	0.88 ± 0.34
	2	3.27 ± 0.15	2.43 ± 0.28	0.83 ± 0.31
	3	3.07 ± 0.18	2.40 ± 0.31	0.68 ± 0.36
June	1	3.29 ± 0.12	2.36 ± 0.27	0.94 ± 0.31
	2	3.19 ± 0.18	2.35 ± 0.28	0.86 ± 0.31
	3	2.92 ± 0.20	2.26 ± 0.31	0.69 ± 0.36

**Table 3:** Results of application of nonparametric tests of the significance of changes in stroke and phase parameters. The entries indicate the presence (1) or absence (0) of significant changes at a *p*-value threshold of 0.05 for datasets of size n=7 (the group of participants), reported in the format “for time duration/for standard deviation.” The exact *p*-values are provided in the Supplementary Materials.

Rank-sum test of ensemble-based changes				
**Stage**	**Grade**	**Full stroke**	**Phase 1**	**Phase 2**
March	1	0/0	1/1	1/1
↓	2	0/0	1/1	0/0
April	3	0/0	1/1	1/1
April	1	0/1	1/1	1/0
↓	2	0/0	1/1	1/0
June	3	0/0	0/1	1/1
March	1	0/0	0/0	0/0
↓	2	0/0	0/1	0/1
June	3	0/0	1/0	0/0
Wilcoxon signed rank test				
**Stage**	**Grade**	**Full stroke**	**Phase 1**	**Phase 2**
March	1	0/0	1/1	1/1
↓	2	0/0	0/0	1/0
April	3	0/0	0/0	1/1
April	1	0/1	1/1	1/0
↓	2	0/0	0/1	1/0
June	3	0/0	0/0	0/0
March	1	0/0	1/0	0/0
↓	2	0/0	0/0	0/0
June	3	0/0	0/0	0/0

**Table 4:** Numerical characteristics of the training load during the study period averaged over the whole group of participants, presented as mean value ± standard deviation. Additionally, the percentage distribution between the two mesocycles for the mean value of each type of activity is reported in parentheses.

Training Means	Mesocycle 1	Mesocycle 2
Number of training sessions	26 ± 0.81 (28.9%)	64 ± 0.72 (71.1%)
Strength exercises, kg	74,800 ± 260 (52.0%)	69,000 ± 785 (48.0%)
Rowing on an indoor rower, continual, m	54,999 ± 646 (35.9%)	98,000 ± 1080 (64.1%)
Rowing on an indoor rower, interval, m	15,080 ± 30 (93.1%)	1100±13 (6.8%)
On-water rowing, continual, m	81,000 ± 910 (28.8%)	200,000 ± 2200 (71.2%)
On-water rowing, interval, min	98 ± 1.4 (28.7%)	243±2.7 (71.3%)

## Data Availability

Additional numerical data are provided in the Supplementary Materials at Supplementary Materials containing an Excel spreadsheet comprising sheets with (a) tables containing the stroke’s mean time supplied with the standard uncertainty for all participants, grades of power loads, and stages of measurement sessions; (b) *p*-values determined by the Rank-sum and Wilcoxon tests; (c) detailed information on the specific distribution of each type and volume of exercise across the days of each month in the training mesocycles. Raw MEMS sensor records and the MATLAB code used to generate all figures are available on GitHub at https://github.com/postnicov/Mems_rowing_data (accessed on 20 May 2020).

## Supplementary Materials

The following supporting information can be downloaded at https://www.mdpi.com/article/10.3390/sports14050214/s1. An Excel spreadsheet with sheets: Stroke period; *p*-values (rank-sum); *p*-values (Wilcoxon); Structure of trainings.
